# Changes in Circulating BDNF in relation to Sex, Diet, and Exercise: A 12-Week Randomized Controlled Study in Overweight and Obese Participants

**DOI:** 10.1155/2019/4537274

**Published:** 2019-11-03

**Authors:** M. Glud, T. Christiansen, L. H. Larsen, B. Richelsen, J. M. Bruun

**Affiliations:** ^1^Department of Nutrition, Exercise and Sports, University of Copenhagen, Frederiksberg, Denmark; ^2^Department of Endocrinology and Internal Medicine (MEA), Aarhus University Hospital, Aarhus, Denmark; ^3^Medical Department M, Randers Regional Hospital, Randers, Denmark

## Abstract

Circulating BDNF is higher in women than in men and suggested to be affected by changes in food intake, body weight, and exercise. The purpose of this study was to compare BDNF concentrations in women and men during a 12-week weight loss intervention. Using a previously published 12-week randomized study, serum BDNF was assessed at baseline and after 12 weeks using an enzyme-linked immunosorbent assay method. Fifty overweight or obese but healthy individuals (26 women, mean age of 36.4 ± 7.9 years; 24 men, mean age of 38.0 ± 5.9 years) were included and allocated into three groups: exercise-only (EXO; 12 weeks of aerobic exercise and isocaloric diet), diet-only (DIO; 8 weeks of very low energy diet (VLED 600 kcal/day) followed by a 4-week weight maintenance diet), or diet and exercise (DEX; 12 weeks of aerobic exercise in parallel with 8 weeks of VLED (800 kcal/day) followed by a 4-week weight maintenance diet). At baseline, BDNF levels were 25% higher in women compared to men (*p*=0.006). Body weight was reduced in all intervention groups (*p* < 0.006). Exercise (EXO group) induced a 22% reduction in circulating BDNF in men (*p*=0.037) and women (*p*=0.080). In the DIO and DEX groups, a significant reduction in BDNF levels (29.9%; *p*=0.035 and 32.5%; *p*=0.003, respectively) was observed in women but not in men. In conclusion, circulating BDNF was significantly changed by diet alone or combined with exercise in women and only by exercise alone in men. This suggests that changes in circulating BDNF depend on weight loss methods (diet/exercise) as well as sex.

## 1. Introduction

Recent evidence has suggested an association between obesity and impaired cognitive functions [1]. In line with this, weight loss using exercise and diet has among other beneficial health-related effects been shown to improve cognitive functions [2]. Several of the biological mechanisms behind these findings are still unknown. However, there is accumulating evidence that brain-derived neurotrophic factor (BDNF) is involved in mediating the benefits of exercise and reduced food intake on cognitive functions [3, 4]. BDNF is a member of the nerve growth factor- (NGF-) related family expressed in the central nervous system (CNS) and peripheral nervous system (PNS) [5]. BDNF has a central role in neuronal outgrowth, differentiation, neuronal repair, and synaptic connection [6]. Further, loss-of-function mutations in the BDNF receptor (high-affinity tropomyosin-related kinase B (TrkB)) can cause diseases, such as depression and neurodegenerative disorders as well as obesity and eating disorders [3, 5, 7]. Sex differences have been shown to affect circulating BDNF [8, 9], with higher circulating BDNF in women, compared with men [10].

A high level of circulating BDNF has been linked to a healthy lifestyle and a low level of BDNF has been associated with metabolic risk factors and eating disorders [3, 11]. In recent studies, conflicting data have modified the associations with low BDNF in overweight individuals [12–14]. Research is needed to clarify the BDNF levels in overweight induced by different factors according to sex. No randomized studies have investigated the combined effect of diet and exercise in overweight on circulating BDNF levels, according to sex.

Due to conflicting results in circulating BDNF, we hypothesized that BDNF levels after weight loss will be significantly different according to sex. The aim of this analysis was to investigate if weight loss in overweight and obese participants, induced by a 12-week intervention of either aerobic exercise, energy-restricted diet, or a combination of both, would affect circulating BDNF and if sex had any effects that would modify the weight loss effect.

## 2. Subjects and Methods

### 2.1. Participants

The primary study is described in detail in the study of Christiansen et al. [15]. In this study, we included 26 women (mean age of 36.4 ± 7.9 years) and 24 men (mean age of 38.0 ± 5.9 years) who completed the primary study and who had serum available for the BDNF analyses. The participants were weight-stable (±2 kg of current body weight), were physically able to complete the exercise programme, and received no medication affecting metabolism. The women included were fertile and in different phases of their menstrual cycle during blood sampling and BDNF assessment; however, given the long intervention (12 weeks) and randomization, the effect of the hormonal changes during the study is considered to be of minor importance. Participants were excluded if they were diagnosed with cardiovascular disease, were diagnosed with type 2 diabetes (T2D), or were pregnant. All gave written informed consent before the participation.

The participants were randomized to a 12-week intervention consisting of either aerobic exercise (EXO), energy-restricted diet (DIO), or a combination of both (DEX). Baseline data were collected at first day of the 12-week intervention.

### 2.2. Exercise

In the EXO and DEX groups, the exercise consisted of supervised aerobic exercise three times per week with a duration of 60–75 min per training session, an estimated energy expenditure of 500–600 kcal per session, and an intensity of 70% of heart rate reserve [16].

### 2.3. Diet

In the DIO and DEX groups, the diet consisted of a liquid very low energy diet (VLED; Nupo, Copenhagen, Denmark) of 600 or 800 kcal/day, respectively (49.6 E% protein, 35.1 E% carbohydrates, and 15.2 E% fat) for 8 weeks followed by a weight maintenance diet for 4 weeks [15]. The weight maintenance diet was determined by estimating resting energy expenditure multiplied by a factor of 1.5 for subjects in the DIO group and 2.5 in the DEX group. In the EXO group, dietary advice was given to maintain an isocaloric diet for the duration of the intervention.

### 2.4. BDNF Assay

The Quantikine ELISA Human Free BDNF immunoassay (DBD00, R&D Systems, Abingdon OX14, UK) was used for the BDNF analysis according to the manufacturers' instructions. Serum was diluted 1 : 20 for the analyses. The intra- and interassay CV was 5.0% and 11.3%, respectively, and all samples were tested in doublet to minimize the interassay variability.

### 2.5. Statistical Analysis

Baseline data are presented as mean ± standard deviation (SD), and changes from baseline to week 12 are presented as mean with 95% confidence interval (CI). Normality of data was tested with the Shapiro–Wilk test. Independent *t*-test and Mann–Whitney *U* test were used to test for sex difference.

Pearson and Spearman correlations were performed to investigate correlations between BDNF, anthropometric, metabolic, and adipose tissue markers in relation to gender. For any significant correlation, linear regression was performed to test for predictors for BDNF. Paired-sample *t*-tests and Wilcoxon test were performed to investigate any significant change from baseline to week 12.

One-way ANOVA was performed to test for interactions between women and men.

Two-way ANOVA was performed to test for interactions between sex and intervention groups.

*p* values below 0.05 were considered statistically significant. All statistical analyses were performed with the statistical software package SPSS (Chicago, IL, USA).

## 3. Results

The analyses included 26 women, who were 36.4 ± 7.9 years and had a mean BMI of 35.7 ± 3.1 kg/m^2^, and 24 men, who were 38.0 ± 5.9 years and had a BMI of 32.3 ± 2.6 kg/m^2^.

**Figure 1:**
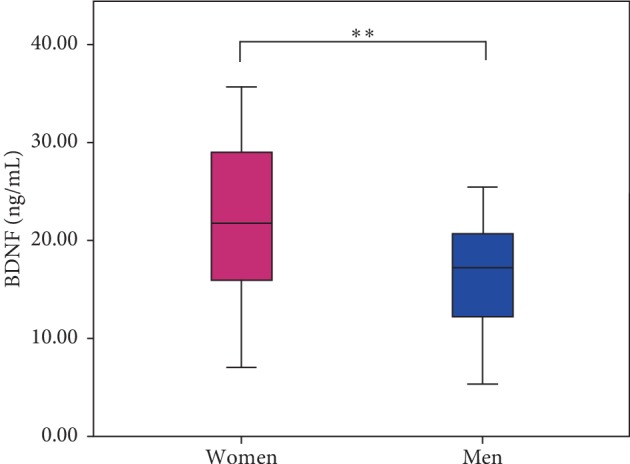
Baseline serum BDNF. Boxplot of average (±standard error) of serum BDNF levels in women (21.7 ± 7.5 ng/mL) and men (16.3 ± 5.7 ng/mL). Significant difference (*p*=0.006) is marked with two stars. Independent *t*-test was used to test for significant sex difference.

At baseline, serum BDNF concentrations were higher in women, 21.7 ± 7.5 ng/mL, compared to men, 16.3 ± 5.7 ng/mL (*p* < 0.01; Figure 1).

In men and women, weight decreased in all intervention groups with the largest weight loss for men in the DIO group with −14.3 ± 2.9 kg (*p* < 0.001) and for women in the DEX group with −11.4 ± 5.2 kg (*p* < 0.001, Table 1). Men had the largest chance in weight loss and BMI across all groups: EXO, DEX, and DIO (Table 1).

In the EXO group, circulating BDNF decreased by 22.1% in men (*p* < 0.05) and 22.4% in women (*p*=0.1, Table 2). In the DIO group, circulating BDNF decreased by 29.9% in women (*p* < 0.05), but no significant effect was observed in men (Table 2). In the DEX group, circulating BDNF decreased by 32.5% in women (*p* < 0.01), but no significant effect was observed in men (Table 2). When comparing the three groups by ANCOVA, we found no significant difference in the change in BDNF levels (Table 2).

In men, insulin decreased significantly in the DIO group (*p* < 0.05), and in women, insulin decreased significantly in the DIO and DEX groups (*p* < 0.05, *p* < 0.05, Table 1).

**Figure 2:**
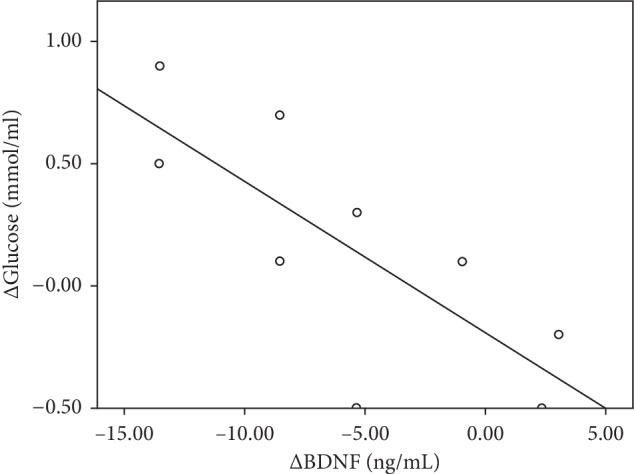
Linear regression of ΔGlucose and ΔBDNF in men in the EXO group. Decrease in serum ΔBDNF was associated with decreased ΔGlucose: *R*^2^ = 0.585, *p*=0.016.

In men and women, glucose levels were significantly decreased in the DIO group (*p* < 0.05, *p* < 0.01, Table 1). For men in the EXO group, there was a positive correlation between ΔBDNF and ΔGlucose (*p* < 0.05, Figure 2).

## 4. Discussion

At present, only a few human studies have investigated the impact of sex and a combination of exercise and diet on circulating BDNF. The present investigation is to our knowledge the first long-term randomized exercise and diet study to demonstrate the sex-dependent effect on serum BDNF in healthy overweight or obese subjects.

Overall, and in accordance with other studies [10, 17, 18], we observed ∼25% higher serum BDNF levels in women compared to men, confirming that circulating BDNF is sex dependent. It has been shown previously that women have a higher expression of BDNF in several brain regions [18, 19], a difference that may be explained by estrogen-induced effects on BDNF signaling pathways [20] and an estrogen-specific effect on BDNF levels found in animal studies [21]. In line with these hormonal effects, women have been shown to have higher circulating BDNF levels in the last phase of their menstrual cycle compared to the first phase [22]. These findings suggest that gonadal hormones could influence the sex difference found in circulating BDNF levels.

Aerobic exercise was in our study found to reduce serum BDNF levels significantly with 22% in men. Most of other current studies have not investigated the effects of sex differences and exercise but analyzed either men or women. However, rodent studies have found higher BDNF mRNA expression and circulating BDNF levels after 5 months of voluntary wheel running in males compared to female mice [23] In humans, and similar to our findings, Damirchi et al. showed a decrease in circulating BDNF in men after aerobic exercise [24], and the meta-analysis of 29 studies by Szuhany et al. agreed on an increase in BDNF after exercise in men, but not in women [4]. Although we found the same relative change in circulating BDNF in women and men, other studies have observed sex differences in the BDNF response related to the intensity of the exercise performed, where high intensity has been found to cause the largest sex difference [25, 26]. These findings could indicate that men are able to obtain higher BDNF levels with increasing intensity of the exercise, which may be explained by the relatively higher muscle mass in men, acknowledging that BDNF may play a role as a regulator of metabolism in skeletal muscle in humans [27]. In addition, a recent study found that low adipose tissue mass was associated with increased BDNF levels in male mice [28]. This sex difference in relation to adipose tissue mass may contribute to some of the sex differences observed in our study after aerobic exercise [29]. A recent study by Golden et al. with obese adolescents showed no sex-dependent effect of either resistance or aerobic exercise [30]. In contrast, circulating BDNF levels increased in women above 45 years after 6 months of aerobic exercise, whereas levels decreased in men [31]. These findings, with age-related differences, could indicate that age has an impact on the sex difference found in BDNF levels after aerobic exercise. Further studies are needed to investigate the association between circulating BDNF levels, exercise, sex, and the influence of age.

We found a significant 30% reduction in circulation BDNF levels in women after the VLED-induced weight loss. In rodents, BDNF expression in the ventromedial hypothalamus has been shown to be regulated by nutritional status [32] and to reduce glucose levels after a high-fat diet [33, 34]. Additionally, sex-specific research in rodents found lower glucose and higher BDNF expression in women compared to men after a high-fat diet [35] In line with these findings, our human study found similar changes in glucose (Table 2), and in men, a significant correlation between change in glucose and change in BDNF after exercise was found. The abovementioned findings may suggest that BDNF levels are related to changes in energy balance including changes in glucose and insulin levels. Whether the observed sex differences in BDNF in our study are caused by sex differences related to glucose metabolism is not possible to determine from the current literature.

The findings in the present study confirm the findings in other human studies with a reduction in circulating BDNF in women after weight loss. Merhi et al. [12] showed a ∼50% decrease in circulating BDNF levels in 18 morbidly obese women 3 months after bariatric surgery and a 12.6% decrease in BMI. In contrast, Harvie et al. [36] found no change in serum BDNF after either intermittent or continuous energy restriction in overweight women. In that study, the intervention with a 25% continuous energy restriction over 6 months resulted in a 3.5% weight loss [36], opposed to the 10% weight loss found in our study where participants followed a VLED for 8 weeks. Changes in body weight in humans have been shown to affect BDNF levels [9, 37], and the present findings suggest that the effect of weight loss on circulating BDNF might depend on the level of energy restriction. Further research in that potential dose-response relationship is needed in order to understand the relations between BDNF levels and energy restriction.

The combination of aerobic exercise and VLED showed a highly significant 32% decrease in circulating BDNF in women with no change in men. The present study is the first human study to combine exercise and diet in relation to circulating serum BDNF levels in healthy overweight or obese female and men. However, animal studies have previously investigated the combined effect of diet and exercise on BDNF levels. Wu et al. demonstrated that dietary supplement of docosahexaenoic acid combined with voluntary exercise increased BDNF levels in rats [38]. Additionally, voluntary exercise has been found to reverse the effects of high-fat diet on circulating BDNF in female rats [39]. This suggests that the effect of diet can be enhanced by the addition of exercise and might overrule the negative effects of a high-fat diet.

The strength of the present study is the randomized design, sex-segregated analysis, monitored diet, and supervised exercise session [15]. There are also some limitations to the study, including the small group of each sex. There is an ongoing discussion in the literature on the choice of serum vs. plasma for the measurement of circulating BDNF. It has been shown that exercise influenced serum BDNF levels more than the plasma levels [40, 41] Studies have suggested that serum presents a more complete amount of circulating BDNF levels compared with plasma, since BDNF is picked up by platelets from the circulating BDNF [42]. However, a recent study showed high reliability in serum measurements of BDNF [43].

## 5. Conclusion

In this secondary analysis, both diet and diet together with exercise changed circulating BDNF levels significantly in women only. Exercise alone altered circulating BDNF in men but only showed a trend in women. When comparing the additional effects of energy restriction in women on circulating BDNF, it would be of interest in future studies to investigate if a weight loss (or energy restriction) threshold exists. Additionally, it would be of interest to investigate if the lifestyle-induced changes in peripheral BDNF levels are mirrored by changes in BDNF levels in the central nervous system.

## Figures and Tables

**Table 1:** Baseline and change (week 0–week 12) characteristics in EXO, DEX, and DIO according to sex.

	EXO				DIO				DEX			
	Women	*p*	Men	*p*	Women	*p*	Men	*p*	Women	*p*	Men	*p*
*N*	7		9		8		6		11		9	
Age	35.6 ± 8.8		38.8 ± 5.4		34.6 ± 7.0		37 ± 5.9		38.3 ± 8.3		37.8 ± 6.8	
Weight (kg)												
Week 0	99.4 ± 6.6		101.8 ± 11.0		106.5 ± 10.2		105 ± 5.9		101.9 ± 17.2		111.4 ± 12	
Week 12	96.0 ± 6.2		97.1 ± 9.3		95.9 ± 9.8		90.7 ± 6.2		90.5 ± 14.4		98.0 ± 10.0	
Change	3.4 ± 2.2	0.006	−4.7 ± 2.3	≤0.001	−10.6 ± 2.8	0.012	−14.3 ± 2.9	≤0.001	−11.4 ± 5.2	≤0.001	−13.3 ± 5.2	≤0.001

BMI (kg/m^2^)												
Week 0	35 ± 1.3	0.006	31.2 ± 2.9		37.2 ± 2.7	0.005	32.4 ± 2.4		35.1 ± 3.9		33.4 ± 2.1	
Week 12	33.8 ± 1.3		29.8 ± 2.5		33.5 ± 2.6		28.0 ± 2.6		31.2 ± 2.9		29.5 ± 2.4	
Change	−1.1 ± 0.7	0.005	−1.4 ± 0.7	≤0.001	−3.7 ± 1.0	≤0.001	−4.4 ± 0.8	≤0.001	−3.9 ± 1.7	0.003	−4.0 ± 1.3	≤0.001

Waist (cm)												
Week 0	105 ± 5.5		104.2 ± 6.5		107.1 ± 6.9		110.2 ± 6.2		106.7 ± 12.3		113.2 ± 5.4	
Week 12	99.0 ± 4.8		98.8 ± 7.0		97.1 ± 7.0		97.5 ± 8.5		95.5 ± 10.9		99.7 ± 7.5	
Change	−6.0 ± 3.5	0.04	−5.4 ± 3.4	0.001	−9.5 ± 3.6	≤0.001	−12.7 ± 3.6	≤0.001	−11.2 ± 5.3	≤0.001	−13.6 ± 4.0	≤0.001

Glucose (mmol/l)												
Week 0	5.5 ± 0.7		5.6 ± 0.3		5.4 ± 0.6		5.5 ± 0.4		5.5 ± 0.5		5.7 ± 0.3	
Week 12	5.6 ± 0.3		5.7 ± 0.6		4.8 ± 0.4		5.3 ± 0.3		5.4 ± 0.5		5.5 ± 0.6	
Change	0.1 ± 0.6	0.575	0.2 ± 0.5	0.376	−0.6 ± 0.4	0.004	−0.2 ± 0.6^*∗*^	0.012	−0.1 ± 0.3	0.104	−0.3 ± 0.5	0.122

Insulin (pg/l)												
Week 0	61.9 ± 36.1		56.8 ± 34		76.4 ± 27.8		83.9 ± 39.5		90 ± 40		85.5 ± 44.2	
Week 12	57.9 ± 38.8		52.8 ± 21.1		51.4 ± 22.0		61.6 ± 23.6		50.5 ± 25.9		64.4 ± 31.0	
Change	−3.9 ± 38.8	0.799	−9.2 ± 29.6	0.410	−25.1 ± 24.6	0.024	−30.6 ± 21.2	0.032	−39.5 ± 45.1	0.016	−21.0 ± 47.7	0.222

HOMA												
Week 0	2.2 ± 1.2		2 ± 1.2		2.7 ± 1.3		3.0 ± 1.4		3.2 ± 1.5		3.2 ± 1.7	
Week 12	2.1 ± 1.4		2.0 ± 1.0		1.6 ± 0.7		2.1 ± 0.8		1.7 ± 0.9		2.3 ± 1.3	
Change	2.1 ± 1.4	0.878	−0.2 ± 1.1	0.564	−1.1 ± 0.9	0.012	−1.2 ± 0.82	0.033	−1.5 ± 1.7	0.017	−0.8 ± 1.9	0.231

Data are presented as mean ± SD. BMI: body mass index; HOMA: homeostatic model assessment; BDNF: brain-derived neurotrophic factor; *p*: *p* value. Paired-sample *t*-test was performed to test for significant change from baseline to week 12. Independent *t*-test was performed to test for sex difference. Mann–Whitney *U* test and Wilcoxon rank test were used for nonparametric data.

**Table 2:** Baseline, week 12, change, and percentage change values for BDNF in EXO, DIO, and DEX according to sex.

	EXO				DIO				DEX			
	Female	*p*	Male	*p*	Female	*p*	Male	*p*	Female	*p*	Male	*p*
BDNF (ng/mL)												
Baseline	21.8 ± 6.7		17.8 ± 6		23.2 ± 8.9		16.9 ± 6.4		20.6 ± 7.3^†^		14.5 ± 5	
Week 12	16.1 ± 4.2		12.1 ± 2.6		15.0 ± 7.3		14.0 ± 5.1		12.8 ± 4.8		12.7 ± 4.9	
Change	−5.7 ± 7.1	0.08	−5.6 ± 6.1^*∗*^	0.026	−8.2 ± 8.9^*∗*^	0.035	−2.9 ± 7.2	0.368	−7.8 ± 6.8^*∗∗*^	0.003	−1.8 ± 6.2	0.409
% change	−22.4 ± 24.3	0.08	−22.1 ± 34.9^*∗*^	0.026	−29.9 ± 31.0^*∗*^	0.035	−4.2 ± 45.6	0.368	−32.5 ± 27.0^*∗∗*^	0.003	−5.8 ± 39.1	0.409

Data are presented as mean ± SD. ^*∗*^Significant difference from baseline to week 12, in absolute values (*p* < 0.005); ^*∗∗*^significant difference from baseline to week 12, in absolute values (*p* < 0.001). BMI: body mass index; HOMA: homeostatic model assessment; BDNF: brain-derived neurotrophic factor; *p*: *p* value. Paired-sample *t*-test was performed to test for significant change from baseline to week 12. Independent *t*-test was performed to test for sex difference.

## Data Availability

The data used to support the findings of this study are available from the corresponding author upon request.
